# Bioactivity guided fractionation and hypolipidemic property of a novel HMG-CoA reductase inhibitor from *Ficus virens* Ait

**DOI:** 10.1186/s12944-015-0013-6

**Published:** 2015-03-04

**Authors:** Danish Iqbal, M Salman Khan, Mohd Sajid Khan, Saheem Ahmad, Md Sarfaraj Hussain, Mohd Ali

**Affiliations:** Department of Biosciences, Clinical Biochemistry & Natural Product Research Lab, Integral University, Lucknow, 226026 India; Department of Pharmacy, Integral University, Lucknow, 226026 India; Department of Pharmacognosy & Phytochemistry, Faculty of Pharmacy, Jamia Hamdard, New Delhi, 110062 India

**Keywords:** Ficus virens, Hyperlipidemia, Antioxidant, HMG-CoA reductase, Molecular Docking, Inhibitor

## Abstract

**Background:**

The current perspective for the search of 3-hydroxy-3-methyl-glutaryl-coenzyme A (HMG-CoA) reductase inhibitor has been shifted towards a natural agent also having antioxidant property. Thus, this study was intended to isolate and identify the bioactive compounds from methanolic extract of *Ficus virens* bark (FVBM) and to evaluate their antioxidant, HMG-CoA reductase inhibitory and hypolipidemic activity.

**Methods:**

Bioactivity guided fractionation and isolation of bioactive compound from FVBM extract has been done to isolate and characterize the potent HMG-CoA reductase (HMGR) inhibitor with antioxidant activity by using repeated extensive column chromatography followed by spectroscopic methods, including Infrared (IR), ^1^H & ^13^C nuclear magnetic resonance (NMR) and Mass spectrum analysis. The *in vitro* HMGR inhibition and enzyme kinetic assay was determined using HMG-CoA as substrate. In addition, antioxidant activity of the new isolated compound, was measured using 2,2-diphenyl-1-picrylhydrazyl (DPPH) radical scavenging assay and FRAP value. *In-silico* molecular informatics of HMGR enzyme type inhibition and pharmacokinetics data of the new compound was further evaluated through molecular docking and ADME-T studies. Further, *in-vivo* hypolipidemic property of FVBM extract and newly isolated compound was also analyzed in triton-WR 1339 induced rats.

**Results:**

Thereby, we report the discovery of n-Octadecanyl-O-α-D-glucopyranosyl(6′ → 1″)-O-α-D-glucopyranoside (F18) as a novel HMG-CoA reductase inhibitor with strong antioxidant property. This inhibitor exhibited not only higher free radical scavenging activity but also marked HMG-CoA reductase inhibitory activity with an IC_50_ value of 84 ± 2.8 ng/ml. This inhibitory activity concurred with kinetic study that showed inhibition constant (*K*_i_) of 84 ng/ml via an uncompetitive mode of inhibition. The inhibition was also corroborated by molecular docking analysis and *in silico* pharmacokinetics data. The *in vivo* study revealed that administration of FVBM extract (at higher dose, 100 mg/rat) and the inhibitor (1 mg/rat) to Triton WR-1339-induced hyperlipidemic rats significantly ameliorated the altered levels of plasma lipids and lipoproteins including hepatic HMG-CoA reductase activity; this effect was comparable to the effect of standard drug atorvastatin.

**Conclusions:**

The *in vitro, in silico* and *in vivo* results clearly demonstrated the antioxidant potential and therapeutic efficacy of the inhibitor as an alternate drug against hyperlipidemia.

**Electronic supplementary material:**

The online version of this article (doi:10.1186/s12944-015-0013-6) contains supplementary material, which is available to authorized users.

## Background

Cardiovascular disease (CVD) is the major cause of death in developed and developing countries [1,2]. It is well known that three major risk factors for CVD are hypercholesterolemia, smoking and hypertension [3]. Several studies have established that elevated blood cholesterol along with triglyceride (TG) level is a major cause and initial risk factor of atherosclerosis and subsequent CVD [4,5]. Endogenous cholesterol biosynthesis in the liver is mainly controlled by rate limiting enzyme, 3-hydroxy-3-methylglutaryl-CoA (HMG-CoA) reductase, which catalyzes the conversion of HMG-CoA to mevalonic acid [6]. Drugs that lower cholesterol level mainly work by inhibiting the HMG-CoA reductase activity [7,8]. Despite the significant clinical benefits provided by statins [9], many patients do not achieve the recommended low-density lipoprotein (LDL) and high-density lipoprotein (HDL) cholesterol target goals [10]. Moreover, elevated lipid level results in accumulation of LDL in subendothelial space of arteries, where it undergoes through an oxidative modification to form oxidized LDL, which is highly atherogenic [5,11]. Moreover, the use of statins is not preferred in more than 40% of patients, mostly due to the occurrence of several side effects including myalgia, myopathy, liver disease, and rhabdomyolysis in more severe cases [12,13]. Statins in combination with fibrates show significant benefit at higher doses but are also associated with severe side effects [14]. This limits the use of statins and incites a search of new natural drugs to combat hypercholesterolemia as well as cholesterol induced oxidative stress and atherosclerosis.

Medicinal plants are potential sources of therapeutic compounds. Therefore, searching for natural and selective HMG-CoA reductase inhibitors with antioxidant property as an alternative to synthetic drugs is of great interest. One of the promising breakthroughs in the drug discovery is the use of mechanism-based screening for a bioassay guided fractionation such as isolation of mevastatin from *Penicillium citrinum* [15,16]. *Ficus virens* Ait (FV) (Moraceae) has been known traditionally for its medicinal properties, which include its use in the treatment of blood diseases, uterus, burning sensation, hallucination, and unconsciousness [17]. This plant is also known to possess significant amount of phenolic compounds and a potent antioxidant activity [18,19]. In a continuous bid to search new hypolipidemic drug with antioxidant property from plant origin, we have recently demonstrated that among all sequentially extracted fractions of *Ficus virens*, *Ficus virens* bark methanolic extract (FVBM) posses a significant HMG-CoA reductase inhibitory activity along with antioxidant property [20]. On this basis, the present study was premeditated to isolate and characterize the bioactive compounds from FVBM extract, and subsequently to evaluate their antioxidant and HMG-CoA reductase inhibitory activity using *in vitro* and *in silico* approaches. Furthermore, *in vivo* lipid lowering activity and the possible mechanism of action of FVBM extract and the bioactive compound have also been discussed.

## Materials and methods

### Chemical reagents

HMG-CoA reductase assay kit was purchased from Sigma-Aldrich Co. (USA). 2, 2-diphenyl-1-picrylhydrazyl (DPPH), Triton WR-1339, 2,4,6-tri(2-pyridyl)-s-triazine (TPTZ), and silica gel (60–120 mesh) were purchased from HiMedia Laboratories, Mumbai, India. Total cholesterol (TC) and triglycerides (TG) kits was procured from Merck Diagnostic (German). All other chemicals and solvents used in this study were of analytical grade.

### Plant material and extraction

The plant material, fresh stem bark of *Ficus virens* Ait (FVB), was collected from herbal garden of the Department of Pharmacy, Integral University, Lucknow, India. Plant was authenticated by Dr. Tariq Husain of the herbarium division of National Botanical Research Institute, Lucknow, India, and has been deposited in herbarium vide Accession No. 97959. The sequential extraction of FVB was performed to obtain methanolic fraction [20].

### Bioactivity guided isolation and characterization of active compound

The dried residue of FVBM extract was subjected to silica gel (60–120 mesh) column chromatography starting with CHCl_3_/MeOH (98:02, v/v) as eluent, followed by a gradient of increasing methanol percentage (i.e., increasing polarity). Twenty fractions (F1-F20) of 200 ml each were collected and tested for antioxidant and HMG-CoA reductase inhibitory activity as described below. The most bioactive fraction (F18) was subjected to 1D and 2D thin layer chromatography (TLC) in order to check the purity and determination of the structure of the bioactive compound by using the following techniques: infrared (IR), ^1^H and ^13^C nuclear magnetic resonance (NMR), and mass spectroscopy. The electrospray mass spectra were recorded on THERMO Finnigan LCQ Advantage max ion trap mass spectrometer. The samples (10 μl) (dissolved in solvent such as methanol/acetonitrile/water) were introduced into the ESI source through Finnigan surveyor autosampler. The mobile phase was 90:10 Methanol/ACN: H_2_O flowed at the rate of 250 μl/min by MS pump. Ion spray voltage was set at 5.3 KV and capillary voltage at 34 V. Each MS scan was recorded for to 2.5 min and the final spectra were average of over 10 scans at peak in TIC. The IR spectra of the antioxidant compounds were recorded on Perkin-Elmer spectrophotometer version 10.03.06. ^1^H NMR and ^13^C NMR spectra were recorded on BrukerDRX-300, using methanol CD3OD as solvent. 1D and 2D TLC were performed on silica gel 60 F_254_ aluminum plate.

### In vitro antioxidant assays

The DPPH radical scavenging activity was measured according to the standard method of Williams *et al.* [21]. Measurement of ferric reducing antioxidant power (FRAP) of the various fractions (F1-F20) was carried out according to the procedure of Benzie and Strain [22] with some modifications [23].

### *In vitro* cell-free assay and kinetic studies of HMG-CoA reductase

The HMG-CoA reductase assay kit from Sigma-Aldrich (St. Louis, MO, USA) based on the catalytic domain of the human enzyme (recombinant GST fusion protein expressed in *E. coli*) was used, following the manufacturer’s protocol. The kit was used for a rapid screening of all the fractions isolated from FVBM extract and for the identification of the most effective bioactive compound with statin-like activities [20].

In order to determine the mode of inhibition of HMG-CoA reductase enzymatic activity, the NADPH concentration was fixed to 400 μM and HMG-CoA was used in the range of 100–500 μM in the absence and presence of different concentrations (10, 50, 100, 500, 1000 ng/ml) of the fraction showing maximum inhibition. Enzyme kinetic parameters (*K*_m_ and *V*_max_), were evaluated using the non-linear regression method based on Michaelis-Menten equation and the type of inhibition was identified using Lineweaver-Burk plot, and *K*_i_ was determined by Dixon plot [24,25].

### Docking analysis

Crystallographic structures used in this study for HMG-CoA reductase in complex with atorvastatin (PDB ID: 1HWK) was retrieved from the Research Collaboratory for Structural Bioinformatics (RCSB) Protein Data Bank (www.pdb.org). The 3D structures of the ligands were prepared with Chembiodraw Ultra 12.0. Ligand-protein docking was performed using AutoDock4.2 program. This program starts with the ligand in an arbitrary conformation and finds favorable dockings in a protein binding site using the Lamarckian genetic algorithm to create a set of possible conformations [26] to dock pravastatin. The software docked the novel compound (F18) isolated from FVBM extract into the catalytic portion of HMG-CoA reductase enzyme using an empirical scoring function based on the free binding energy [27]_._ After execution of Autodock, several conformations of ligand in complex with the receptor were obtained, which were finally ranked on the basis of binding energy and inhibition constant (*K*_i_). The resulting conformations were visualized in the discovery studio visualizer (version 3.2).

### Absorption, distribution, metabolism, and excretion (ADME) studies

Pharmacokinetic parameters, such as aqueous solubility [28], blood–brain-barrier (BBB) penetration [29], plasma protein binding (PPB) [30], intestinal absorption [31] and cytochrome P450 inhibition [32] for the compound of most potent fraction (F18) and standard, were determined by using Descriptors algorithm in Accelrys Discovery Studio 3.5 software. Standard levels for ADME parameters are listed in Table 1.

**Table 1:** **Standard levels of ADME descriptors from Accelrys Discovery studio 2.5**

**Level**	**Aqueous Solubility Intensity**	**Intestinal Absorption Intensity**	**BBB** ^**a**^ **Intensity**	**PPB** ^**b**^ **% of binding**	**CYP450** ^**c**^ **Value**	**Hepatotoxicity**
0	Extremely low	Good	Very high	<90%	Non inhibitor	Extremely low
1	No, very low	Moderate	High	>90%	Inhibitor	
2	Yes, low	Low	Medium	>95%		
3	Yes, good	Very low	Low			
4	Yes, optimal	Undefined	Undefined			
5	No, too soluble					
6	Unknown					

BBB^a^=Blood Brain Barrier, PPB^b^=Plasma Protein Binding, CYP450^c^=Cytochrome P450, Acceptable range of hydrogen bond acceptor=<10 and hydrogen bond donor=<5.

### *In vivo* hypolipidemic experiment

#### Animals

Male Sprague–Dawley (SD) rats weighing around 100–150 g were procured from Indian Institute of Toxicology Research Center, Lucknow. The study protocol was approved by Institutional Animal Ethics Committee (IAEC) (registration number: IU/Biotech/project/CPCSEA/13/14). The rats (5 per cage) were housed for one week in the animal house at temperature 21–22°C with 12 hour light and dark cycles. The rats were given standard diet and water *ad libitum*.

#### Dose preparation

Sequentially obtained FVBM extracts, the bioactive fraction (F18), and the reference drug atorvastatin were dissolved in 10% dimethyl sulfoxide (DMSO) at different concentrations and were homogenized with saline. The doses of the extracts were selected on the basis of previously published reports [33,34].

#### Induction of hyperlipidemia

Hyperlipidemia was induced in rats by a single intraperitoneal injection of Triton WR-1339 (isooctylpolyoxyethylene phenol) (200 mg/kg body weight) dissolved in 0.9% saline. After one hour of tritonization, the animals were given food and water *ad libitum* [35]. Rats in normal control group were injected with saline only. The rats were divided randomly and equally (5 rats in each group) in groups as illustrated in Table 2. The plant extract, bioactive compound (F18) and atorvastatin suspension was administered through gastric intubation in single doses of 0.5 ml/rat, just after the tritonization; and blood was withdrawn after 18 hours [36].

**Table 2:** **Protocol for the treatment of triton induce hyperlipidemia in rats**

**Group**	**Treatment/route of administration**
NC	Solvent system (vehicle)
HLC	Hyperlipidemic control
FVT-1	Hyperlipidemic + plant extract (FVBM) (50 mg/rat)
FVT-2	Hyperlipidemic + plant extract (FVBM) (100 mg/rat)
CT	Hyperlipidemic + bioactive compound (F18) (1 mg/rat)
AT	Hyperlipidemic + standard (atorvastatin) (1 mg/rat)

#### Collection of blood and plasma

At the end of the experiment, rats in each group were anaesthetized; and blood was collected in heparinized tubes by cardiac puncture. Plasma was separated from blood by centrifugation at 2,500 rpm for 30 min, was aliquoted and stored at either 4 or–20°C for future use.

#### Preparation of liver homogenate

After the experiment, the liver from the rats was promptly excised and chilled in ice-cold saline. After washing with saline, it was blotted and weighed. Each liver was cut into pieces; and 10 g of wet tissue was homogenized with 90 ml of chilled 0.1 M sodium phosphate buffer, pH 7.4, containing 1.17% KCl in a waring blender. The homogenate was centrifuged at 1,000 rpm for 10 min at 4°C, and finally was aliquoted and stored at–20°C.

#### Isolation of plasma LDL and HDL

Isolation of plasma LDL and HDL was carried out as described by Wieland & Seidel [37] and Patsch *et al.* [38], respectively.

#### Determination of plasma triglyceride

Plasma TG was determined by using enzymatic kit (Merck, India) based on glycerol-3-phosphate oxidase peroxides (GPO-POD) method [39]. The very low density lipoprotein-cholesterol (VLDL-C) in plasma was calculated by dividing plasma TG values (mg/dl) by a factor of 5 as described by Friedewald *et al.* [40].

#### Determination of plasma TC, LDL-C and HDL-C

Plasma TC, LDL-C and HDL-C were determined by using cholesterol enzymatic kit (Merck, India) based on cholesterol oxidase phenol aminophenazone (CHOD-PAP) method, and results were expressed as mg/dl.

#### Assay of HMG-CoA reductase activity in the liver homogenate

HMG-CoA reductase enzyme activity in the liver homogenate was estimated indirectly as described by Rao and Ramakrishnan [41]. Fresh 10% tissue homogenate (1 g) was mixed with 9 ml of saline arsenate (0.1% sodium arsenate in physiological saline); and 10 ml of 5% perchloric acid was added. It was kept for 5 min at room temperature and centrifuged at 2,000 rpm for 10 min. One milliliter of the supernatant from each tube was taken out and mixed with 0.5 ml of freshly prepared 1 M aqueous hydroxylamine hydrochloride; whereas, for the assay of HMG-CoA, 0.5 ml of alkaline hydroxylamine hydrochloride was added and mixed. After an incubation of 5 min at room temperature, 1.5 ml of 0.616 M ferric chloride reagent containing 5.2% TCA, prepared in 0.65 N HCl was added and mixed; and absorbance was read at 540 nm against a reagent blank using a Biospectrum-kinetics spectrophotometer (Eppendorf) after 10 min of incubation at room temperature.

### Lactate dehydrogenase (LDH) assay

The LDH release assay was performed as per Ahmad *et al.* [42]. In brief, the platelet rich plasma was obtained from the supernatant resulting from the centrifugation of blood at 200 g at room temperature. F18 (1 mg) and Atorvastatin (1 mg) were incubated with platelet for 2 h and the cytotoxicity of the F18 on platelets was measured by the release of lactate dehydrogenase (LDH) from platelets suspension lysed with 1% Triton X-100 using the commercially available LDH kit (Biomedical Research Services).

### Data analysis

For all assays, samples were analyzed in triplicates, and the results are being expressed as mean ± SD. IC_50_ (50% inhibitory concentration) was calculated by Origin v. 6.0 professional software (Origin Lab Corporation, Northampton, MA, USA), and the results were evaluated using one-way analysis of variance (ANOVA) and two tailed Student’s *t*-test. Statistically significant values were expressed as *p < 0.05, **p < 0.01 and ***P < 0.001.

## Results

In continuation to our previous study that illustrated presence of most potent antioxidant and HMG-CoA reductase inhibitory property in FVBM extract [20], the current one deals with isolation of the active compound through bioassay guided fractionation and its characterization through IR, NMR and mass spectrometry. Besides this *in vivo* hypolipidemic effect of the compound was also evaluated.

### *In vitro* antioxidant property

**Figure 1:**
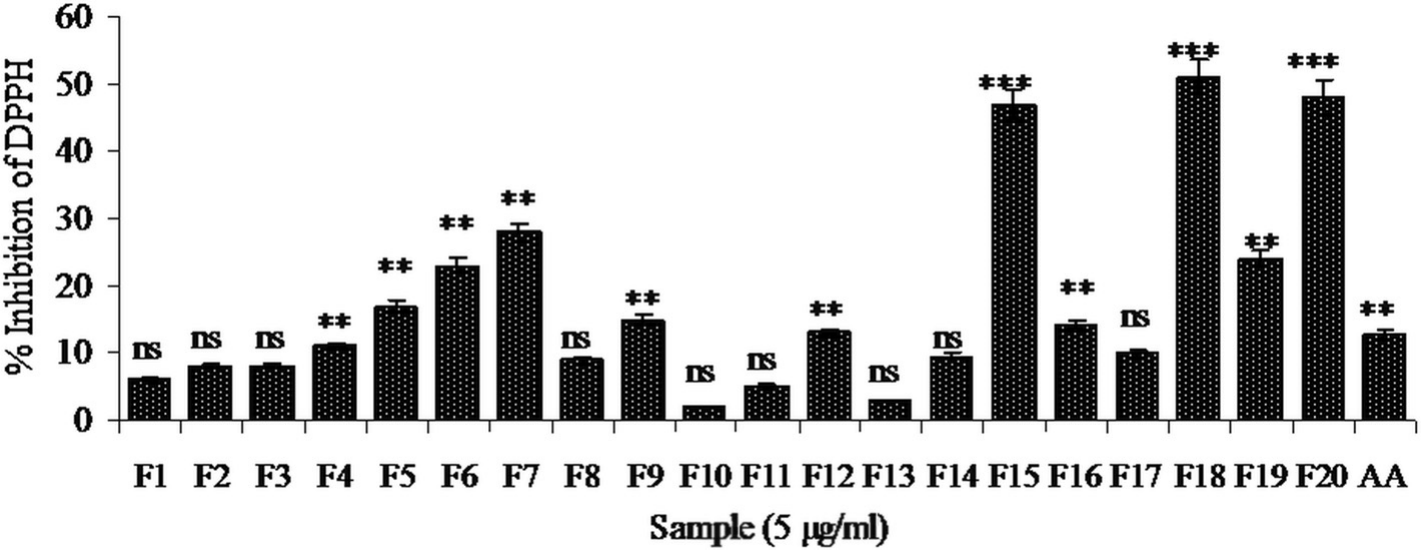
**DPPH free radical scavenging activity of different FVBM fractions (F1-F20) and ascorbic acid (AA).** Values are represented as Mean ± SD (n=3). Non-significant (ns), significantly different at *P < 0.05, **P < 0.01, ***P < 0.001 vs 0 μg/ml.

**Figure 2:**
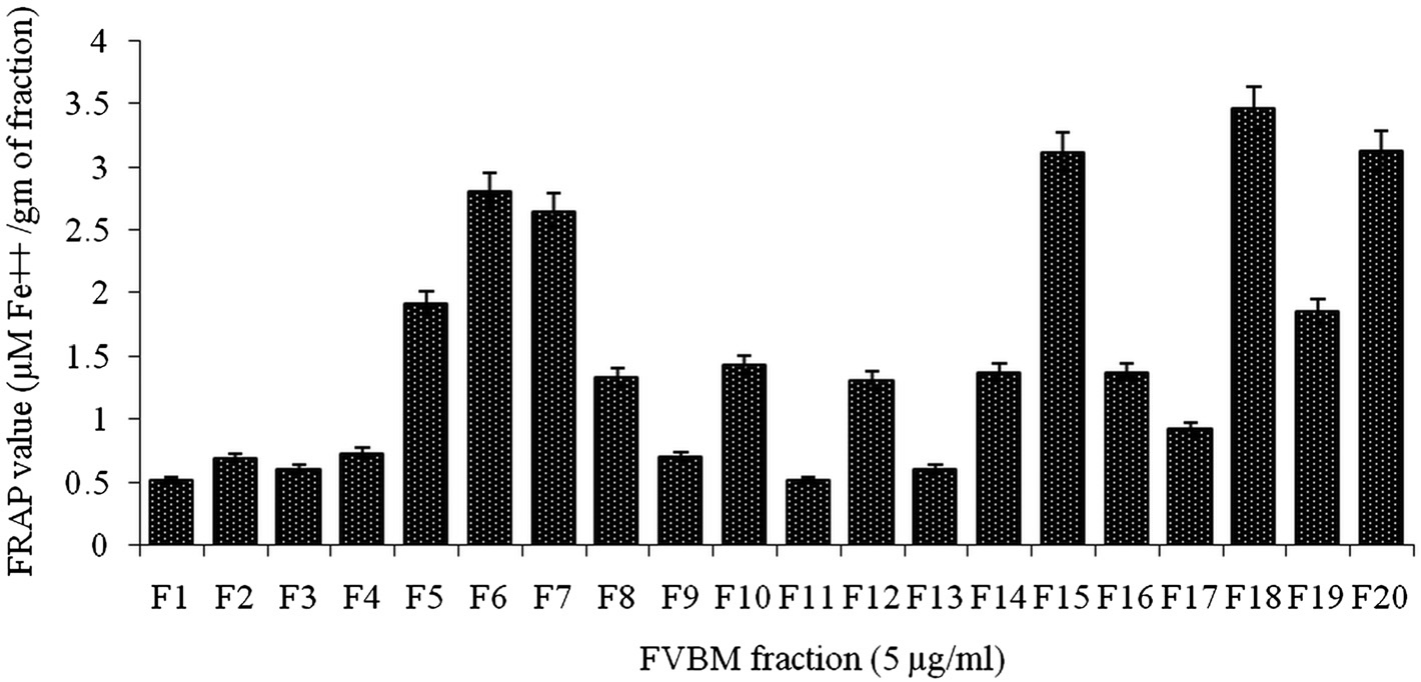
**Ferric reducing antioxidant power of different FVBM fractions.** Values are represented as Mean ± SD (n=3).

Since, the role of free radicals in the pathogenesis of various diseases including atherosclerosis has been well established; the scavenging property of natural agents or drug against free radicals is of utmost importance. Among the eluted FVBM fractions, fraction F18 exhibited significantly higher DPPH radical scavenging activity of 51% at 5 μg/ml (Figure 1), which was much better than standard ascorbic acid value of 12.77% at 5 μg/ml. Moreover, the ability of these fractions to reduce ferric ions into ferrous form, as an indicative of total antioxidant capacity, were also accessed by FRAP assay. Fraction F18 had significantly higher total antioxidant capacity (3.536 μM Fe^2+^/gm) followed by fraction F20 (3.168 μM Fe^2+^/gm), while other fractions had remarkably less activity (Figure 2).

### *In vitro* HMG-CoA reductase inhibitory study

**Figure 3:**
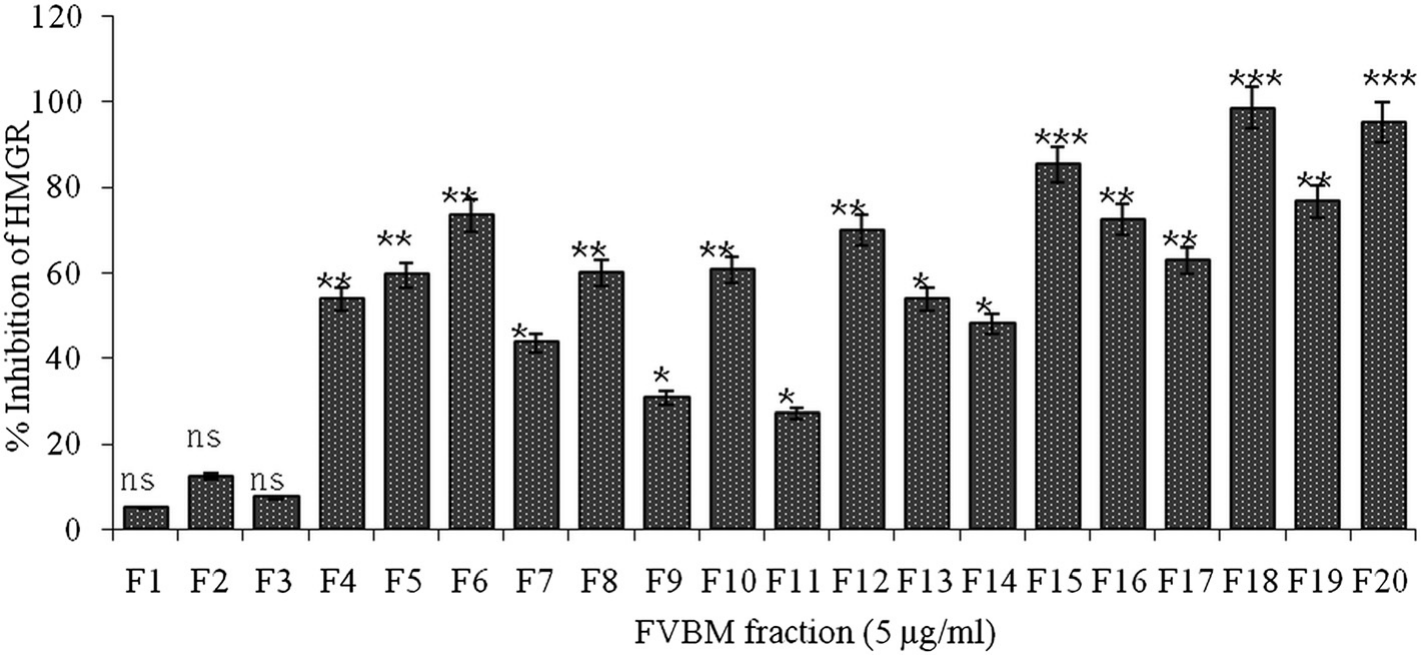
***In-vitro*** **HMG-CoA reductase inhibitory activity of FVBM fractions.** Values are represented as Mean ± SD (n=3). Non-significant (ns), significantly different at *P < 0.05, **P < 0.01, ***P < 0.001 vs 0 μg/ml.

**Figure 4:**
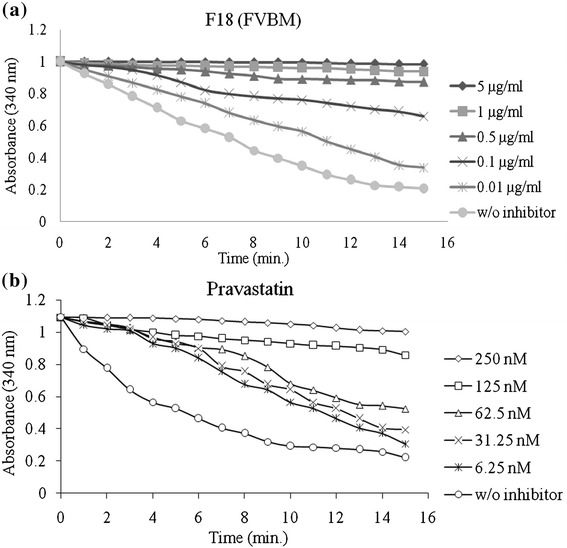
**Spectrophotometric time-scans demonstrating the ability of fraction F18 (n-Octadecanyl-O-α-D-glucopyranosyl (6′ → 1″)-O-α-D-glucopyranoside) (a) and pravastatin (b) to inhibit HMG-CoA reductase activity.**

**Figure 5:**
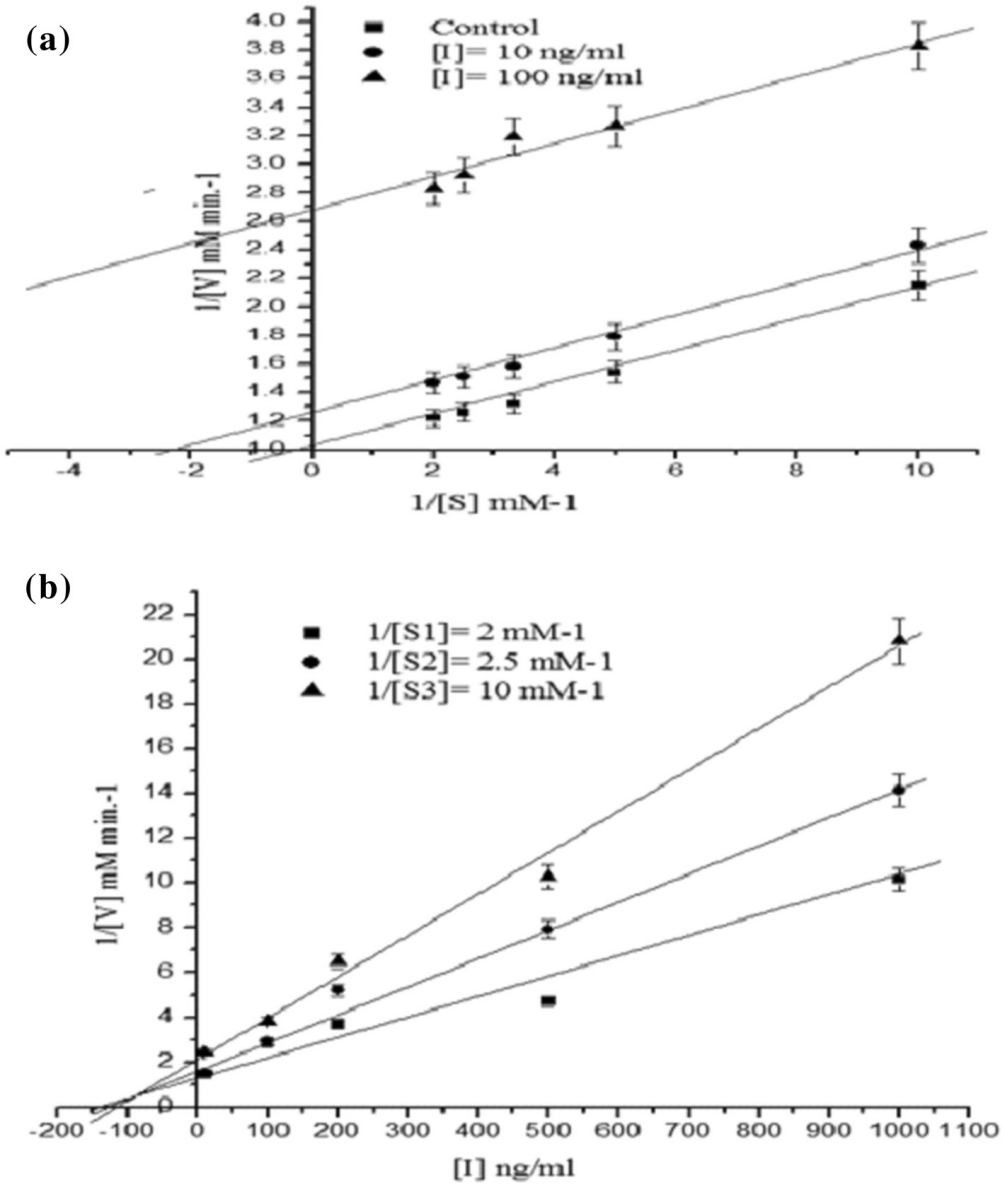
**Lineweaver-Burk double reciprocal plot (a) and Dixon plot (b) of fraction F18 (n-Octadecanyl-O-α-D-glucopyranosyl (6′ → 1″)-O-α-D-glucopyranoside) against HMG-CoA reductase.**

It is well established that cholesterol lowering drugs mainly act by inhibiting the enzymatic activity of HMG-CoA reductase. Therefore, in the present study all the eluted fractions were screened for HMG-CoA reductase inhibitory activity following a previously standardized protocol [20,43]. Among all the eluted fractions, fraction F6, F15, F18, and F20 showed marked HMG-CoA reductase inhibition activity with 73.3%, 85.2%, 98.5% and 98.1% inhibition, respectively, at 5 μg/ml (Figure 3). The fraction F18 due to better antioxidant activity and anti-HMG-CoA reductase property was selected for further study. This fraction has IC_50_ value of 84 ± 2.8 ng/ml, while pravastatin exhibits an IC_50_ value of 70 nM (Figure 4). Furthermore, the mode of inhibition of fraction F18 was also analyzed by double-reciprocal Lineweaver-Burk plot according to Michaelis-Menten kinetics. This fraction demonstrated an uncompetitive mode of inhibition against HMG-CoA reductase (Figure 5a). *K*_i_ value, determined by Dixon plot, of fraction F18 was 84 ng/ml (Figure 5b) and concorded with IC_50_ value, i.e., 84 ± 2.8 ng/ml.

### Identification of the newly isolated bioactive compound

**Figure 6:**
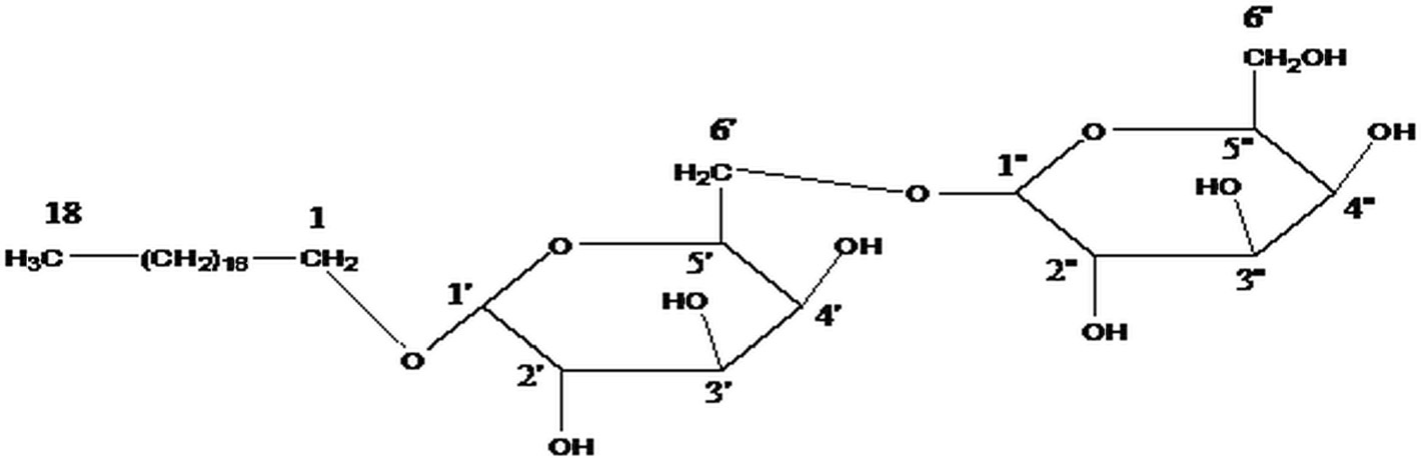
**The structure of bioactive fraction F18 (n-Octadecanyl-O-α-D-glucopyranosyl(6′ → 1″)-O-α-D-glucopyranoside).**

The bioactive fraction F18 was further subjected to IR, ^1^H NMR, ^13^C NMR and mass analysis for its identification and structure determination. The bioactive compound was identified as n-Octadecanyl-O-α-D-glucopyranosyl(6′ → 1″)-O-α-D-glucopyranoside. The ^1^H NMR data showed that this fraction was approximately 95% pure. It gave positive Fehling’s test for glucoside and showed IR absorption bands for hydroxyl groups (3411, 3345, 3211, 3019 cm^−1^), ester function (1626 cm^−1^) and aliphatic chain (756 cm^−1^). On the basis of mass and ^13^C NMR spectra, the molecular ion peak was identified at *m*/z 594 [M]^+^ in consistence with the molecular formula of acyl diglucoside, C_30_H_58_O_11_ (Figure 6). The ^1^H NMR spectrum of F-18-FVBM displayed two one-proton doublets at δ 5.38 (J=4.2 Hz) and 5.11 (J=7.0 Hz) assigned to anomeric H-1′ and H-1′ protons and two two-proton doublets at δ 3.34 (J=6.9, 9.3 Hz) and 3.09 (J=7.5, 6.6 Hz) respectively, other sugar proton from δ 3.96 to 3.30, two two-proton triplet at δ 3.43 (J=6.3 Hz) and 1.99 (J=10.8 Hz) attributed to methylene H_2_-6′ proton adjacent to the ester group, other methylene proton as one proton multiplet from δ 4.63 to 3.44 and two-proton multiplet at δ 1.87, 1.78 and as singlet at δ 1.62 and a three proton triplets at δ 0.87 (J=6.2 Hz) accounted to C-18 primary methyl protons. The ^13^C NMR spectrum of fraction F18 exhibited signals for ester carbons at δ 171.52 (C-1), anomeric carbons at δ 103.27 (C-1′) and 93.32 (C-1″), other sugar carbons between δ 83.39-62.90, methylene carbons from δ 33.88 to 22.55 and methyl carbon at δ 14.61 (C-16). The presence of C-2′ carbon signal in the deshielded region at δ 83.39 indicated attachment of the second sugar unit at C-2′. The absence of any signal beyond δ 4.04 in the ^1^H NMR spectrum and from δ171.52 to 103.27 in the ^13^C NMR spectrum suggested saturated nature of the compound (Additional file 1: Figure S1, Additional file 2: Figure S2, Additional file 3: Figure S3 and Additional file 4: Figure S4). On the basis of this evidence the active compound in fraction F18 was determined as n-Octadecanyl-O-α-D-glucopyranosyl(6′ → 1″)-O-α-D-glucopyranoside (Figure 6).

### *In-silico* study of bioactive compound with HMG-CoA reductase

**Figure 7:**
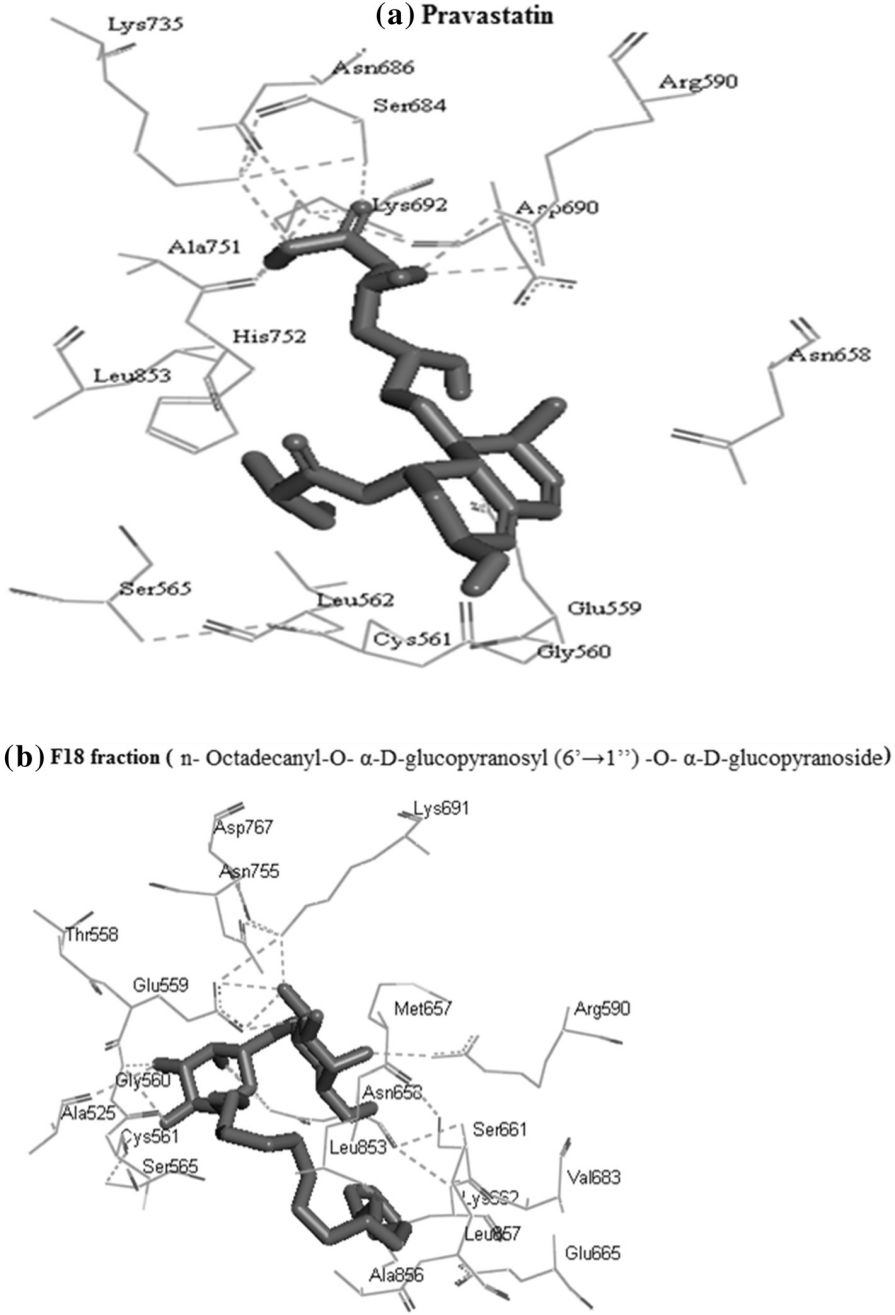
**Binding pattern studies of standard (a) and n-Octadecanyl-O-α-D-glucopyranosyl (6′ → 1″)-O-α-D-glucopyranoside (b) with HMG-CoA reductase.**

To further corroborate our findings, the molecular interaction studies were carried out to find out the plausible ligand-receptor interactions between the inhibitor and HMG-CoA reductase. The docked structures of pravastatin-HMG-CoA reductase were found to be surrounded by Ser^684^, Asp^690^, Arg^590^, Lys^735^ and Glu^559^ amino acid residues, which play important role in stabilizing the complex, whereas n-Octadecanyl-O-α-D-glucopyranosyl(6′ → 1″)-O-α-D-glucopyranoside-HMG-CoA reductase complex involved contributions from residues Asn^755^, Asp^765^, Lys^691^, Glu^559^, Gly^560^, Ala^525^, Arg^590^, which are not from the catalytic domain of HMG-CoA reductase. This observation indicated an uncompetitive mode of inhibition. The non-catalytic site accommodated the compound (Table 3) with a dominant nonelectrostatic energy contribution. The inhibitor showed binding energy of–5.58 Kcal/mol indicating high affinity for the binding site, though pravastatin was a more stronger ligand (ΔG:–6.25 kcal/mol) (Figure 7 and Table 3).

**Table 3:** **Molecular interaction studies of F18 bioactive compound and standards with HMG-CoA reductase**

**S. No.**	**Compound name**	**binding energy ΔG (kcal/mol)**	**Residues involved**
1	n-Octadecanyl-O-α-D-glucopyranosyl(6′ → 1″)-O-α-D-glucopyranoside.	−5.58	ASN755, ASP765, LYS691, GLU559, GLY560, ALA525, ARG590
2	Pravastatin	−6.25	SER684, LYS735, ARG590, ASP690, GLU560, ASN658

### ADME predictions

ADME predictions study of novel compound was done to analyze its pharmacokinetic properties such as aqueous solubility, intestinal absorption, plasma protein binding, blood–brain-barrier penetration, hydrogen bond donor, hydrogen bond acceptor and cytochrome P_450_ inhibition (Table 4). A comparison between these results and the standard values revealed that this bioactive compound has a good aqueous solubility, undefined blood–brain-barrier penetration, low intestinal absorption, <90% plasma protein binding, no inhibition effect on cytochrome P450 and extremely low hepatotoxicity.

**Table 4:** **Predicted ADME profiles for F18 bioactive compound and standard**

**Compound**	**Aqueous Solubility Intensity**	**Intestinal Absorption Intensity**	**BBB Intensity**	**PPB % of binding**	**CYP450 Value**	**Hepatotoxicity**	**A logp 98**	**Hydrogen bond acceptor**	**Hydrogen bond donor**
F18	3	3	4	0	0	0	3.863	11	7
Atorvastatin	2	2	4	1	1	1	4.701	7	4

### LDH release assay

There was no significant increase in LDH released following incubation of blood platelets with either the test drug F18 or the atorvastatin. 4.3% of LDH was released in the incubation medium containing hemoglobin and F18. F18 induced weak LDH leakage suggesting that at concentration of 1 mg F18 was not cytotoxic. Moreover, when compared with the standard, at a concentration of 1 mg there is a released of only 3.7% of LDH in the incubation medium. Around 4% LDH leakage is supposed to be weak in cytotoxicity and hence can be said as non-cytotoxic at this concentration (Table 5).

**Table 5:** **Effect of F18, and atorvastatin on total lactate dehydrogenase released in the incubation medium**

**Incubation time (Min.)**	**Control**	**Atorvastatin (1 mg)**	**F18 (1 mg)**
0	0 ± 0.00	0 ± 0.000	0 ± 0.000
30	0.4 ± 0.30	1.1 ± 0.25	1.3 ± 0.23
60	1.1 ± 0.25	2.0 ± 0.24	2.2 ± 0.35
90	1.6 ± 0.22	3.1 ± 0.31	3.7 ± 0.41
120	2.6 ± 0.24	3.7 ± 0.36	4.3 ± 0.37

Results are presented as mean ± SD.

### *In vivo* hypolipidemic study

The efficacy of FVBM extract (50 and 100 mg/rat), isolated bioactive compound (1 mg/rat) and standard (Atorvastatin, 1 mg/rat) in ameliorating the lipid level was elucidated in Triton WR-1339 induced hyperlipidemic rats after 18 h of treatment. A significant increase in TG (298%), TC (377%) and non HDL-C (592%) levels in Triton WR-1339 induced hyperlipidemic rats was observed in comparison to NC rats (Table 6). After treatment TG, TC & non-HDL-C levels were markedly decreased by 74%, 77% and 83% respectively in the bioactive compound treated group, which was almost comparable to the reduction observed in AT group at the same dose of 1 mg/rat. Furthermore, plasma LDL-C and VLDL-C levels were significantly increased from the value of 36 mg/dl and 12 mg/dl to 280 mg/dl and 48 mg/dl respectively in Triton WR-1339 induced hyperlipidemic rats (Table 7). Whereas, the HDL-C value was significantly reduced by 20% in HLC group, when compared to rats in the control group. After the treatment, a significant decrease of 71%, and 60% in LDL-C, and 86% and 71% in VLDL-C was observed in FVT-1 and FVT-2 groups respectively, when compared to HLC group. Moreover, hyperlipidemic rats treated with bioactive compound (CT) showed marked reduction in LDL-C (85%) and VLDL-C (74%) levels, when compared to HLC group, which is almost comparable to the reduction observed in groups treated with high dose of plant extract (FVT-2: 100 mg/rat) and atorvastatin (1 mg/rat). In contrast the HDL-C level was significantly increased by 19%, 35%, 28% and 29% in FVT-1, FVT-2, CT and AT groups, when compared to HLC control rats. These results demonstrated that the plant extract at higher dose (FVT-2), the bioactive compound and atorvastatin were almost equally effective in ameliorating plasma lipid levels.

**Table 6:** **Effect of FVBM extract, F18 bioactive compound and atorvastatin on plasma triglycerides, total cholesterol and non-HDL-cholesterol in Triton WR-1339 induced hyperlipidemic rats after 18 hours of treatment**

**Group**	**Triglycerides**	**Total cholesterol**	**Non-HDL-cholesterol** ^**†**^
NC	60.45 ± 1.22*	75.86 ± 1.32	47.5 ± 1.17
HLC	240.74 ± 3.36*	361.92 ± 4.86	328.38 ± 3.6
	(+298.24%)^a^	(+377.08%)^a^	(+591.32%)^a^
FVT-1	95.47 ± 2.69*	126.17 ± 2.8	100.24 ± 2.5
	(−60.34%)^a^	(−65.13%)^a^	(−69.47%)^a^
FVT-2	65.42 ± 1.17*	84.25 ± 1.25	53.3 ± 1.19
	(−72.82%)^a^	(−76.79%)^a^	(−83.76%)^a^
CT	62.7 ± 1.48*	84.5 ± 2.25	55.8 ± 1.12
	(−73.95%)^a^	(−76.65%)^a^	(−83.00%)^a^
AT	58.6 ± 1.15*	78.23 ± 1.25	49.6 ± 1.18
	(−75.65%)^a^	(−78.38%)^a^	(−81.85%)^a^

^**†**^For the calculation of non-HDL-C, data is taken from Tables 5 and 6.

^*^Values are mean (mg/dl) ± SD from plasma of 5 rats in each group.

NC, normal control; HLC, triton induced hyperlipidemic control; FVT-1, fed 50 mg FVBM extract/ rat; FVT-2, fed 100 mg FVBM extract/ rat; CT, fed 1 mg F18 bioactive compound/ rat and AT, given 1 mg atorvastatin/rat once.

Significantly different from NC at ^a^p < 0.001.

Significantly different from HLC at ^a^p < 0.001.

**Table 7:** **Effect of FVBM** ***,*** **F18 bioactive compound and atorvastatin on plasma LDL-C, HDL-C and VLDL-C in Triton WR-1339 induced hyperlipidemic rats after 18 hours of treatment**

**Group**	**LDL-C**	**HDL-C**	**VLDL-C**
NC	35.5 ± 1.12*	27.5 ± 0.99	12 ± 0.67
HLC	280.12 ± 4.36*	22.28 ± 0.56	48.26 ± 1.45
	(+646.66%)^a^	(−20%)^a^	(+300.04%)^a^
FVT-1	81.15 ± 2.19*	26.63 ± 0.74	19.09 ± 0.81
	(−71.07%)^a^	(+19.3%)^a^	(−60.41%)^a^
FVT-2	39.5 ± 1.37*	29.7 ± 1.15	13.08 ± 0.64
	(−85.89%)^a^	(+35%)^a^	(−71.40%)^a^
CT	43.26 ± 1.25*	28.2 ± 1.14	12.54 ± 0.63
	(−84.55%)^a^	(+28.18%)^a^	(−74.01%)^a^
AT	37.88 ± 1.18*	28.4 ± 1.15	11.72 ± 0.52
	(−86.47%)^a^	(+29.09%)^a^	(−75.71%)^a^

^*^Values are mean (mg/dl) ± SD from LDL-C, HDL-C and VLDL-C, isolated from plasma of 5 rats in each group.

NC, normal control; HLC, triton induced hyperlipidemic control; FVT-1, fed 50 mg FVBM extract/rat; FVT-2, fed 100 mg FVBM extract/rat; CT, fed 1 mg F18 bioactive compound/rat and AT, given 1 mg atorvastatin/rat once.

Significantly different from NC at ^a^p < 0.001.

Significantly different from HLC at ^a^p < 0.001.

HDL-C/TC, HDL-C/LDL-C, TC/HDL-C and LDL-C/HDL-C ratios calculated from the data presented in Tables 6 and 7 are shown in Table 8. A significant decrease of 5.89 and 9.74 fold in HDL-C/TC and HDL-C/LDL-C ratios was observed; whereas, a marked increase in TC/HDL-C and LDL-C/HDL-C ratios was observed in HLC group in contrast to the normal ratios. After the treatment, the decrease in HDL-C/TC and HDL-C/LDL-C ratio was significantly prevented and increased to 3.43, 4.13 and 5.73, 9.45 fold, respectively, in FVT-1 and FVT-2 group. Furthermore, atorvastatin and bioactive compound treated rats also exhibited marked increase of 5.42, 8.19 and 5.90, 9.43 fold in HDL-C/TC and HDL-C/LDL-C ratio. An opposite pattern was observed in TC/HDL-C and LDL-C/HDL-C ratios. These ratios were significantly increased by 5.88 and 9.73 fold in HLC group, when compared to the normal value of 2.75 mg/dl and 1.29 mg/dl, respectively. All the treated groups showed a significant reduction in TC/HDL-C and LDL-C/HDL-C ratios when compared to the ratios of HLC group. These results indicated a significant restoration of these ratios close to the normal values, which in turn indicated the normalization of cholesterol level associated with the studied lipoproteins.

**Table 8:** **Effect of FVBM, F18 bioactive compound and atorvastatin on the ratios of plasma HDL-C/TC, HDL-C/LDL-C, TC/HDL-C and LDL-C/HDL-C in Triton WR-1339 induced hyperlipidemic rats after 18 hours of the treatment**

**Group/Ratio** ^**†**^	**HDL-C/TC**	**HDL-C/LDL-C**	**TC/HDL-C**	**LDL-C/ HDL-C**
NC	0.3625 ± .025*	0.7746 ± 0.088	2.758 ± 0.105	1.2909 ± 0.0576
HLC	0.0615 ± 0.003*	0.0795 ± 0.0074	16.244 ± 0.76	12.5727 ± 0.65
	(−5.89 f)^a^	(−9.74 f)^a^	(+5.88 f)^a^	(+9.73 f)^a^
FVT-1	0.2110 ± 0.0048*	0.3281 ± 0.0075	4.737 ± 0.094	3.0473 ± 0.086
		(+4.13 f)^a^	(−3.43 f)^a^	(−4.12 f)^a^
	(+3.43 f)^a^			
FVT-2	0.3525 ± 0.0071*	0.7518 ± 0.074	2.836 ± 0.115	1.3299 ± 0.034
		(+9.45 f)^a^	(−5.72 f)^a^	(−9.45 f)^a^
	(+5.73 f)^a^			
CT	0.3337 ± 0.0042*	0.6518 ± 0.015	2.996 ± 0.081	1.5340 ± 0.0691
		(+8.19 f)^a^	(−5.42 f)^a^	(−8.21 f)^a^
	(+5.42 f)^a^			
AT	0.3630 ± 0.0036*	0.7497 ± 0.028	2.754 ± 0.129	1.3338 ± 0.064
		(+9.43 f)^a^	(−5.89 f)^a^	(−9.42 f)^a^
	(+5.90 f)^a^			

^**†**^For the calculation of ratios, data is taken from Tables 5 and 6.

^*^Values are mean (mg/dl) ± SD from plasma of 5 rats in each group.

NC, normal control; HLC, triton induced hyperlipidemic control; FVT-1, fed 50 mg FVBM extract/rat; FVT-2, fed 100 mg FVBM extract/rat; CT, fed 1 mg F18 bioactive compound/rat and AT, given 1 mg atorvastatin/rat once.

‘f’ stands for fold.

Significantly different from NC at ^a^p < 0.001.

Significantly different from HLC at ^a^p < 0.001.

The hepatic HMG-CoA reductase activity in Triton WR-1339 induced hyperlipidemic HLC rats was significantly increased by 3.59 fold when compared to NC rats (Table 9). Administration of plant extract in two different doses resulted in a significant decrease in HMG-CoA reductase activity by 2.06 and 3.50 fold respectively, when compared to HLC value. It is noteworthy that isolated compound exerted better and significant reduction (3.18 fold) than the atorvastatin (2.18 fold), when compared to HLC control group. These results demonstrated that the decrease in cholesterol level of Triton WR-1339 induced hyperlipidemic rats was due to the decline in enzymatic activity of HMG-CoA reductase activity.

**Table 9:** **Effect of FVBM, F18 bioactive compound and atorvastatin on** ***in vivo*** **modulation of hepatic HMG-CoA reductase activity in Triton WR-1339 induced hyperlipidemic rats after 18 hours of the treatment**

**Group**	**HMG-CoA reductase activity** ^**†**^
NC	4.35 ± 0.22^*^
HLC	1.21 ± 0.041^*^
	(+3.59 f)^a^
FVT-1	2.499 ± 0.057^*^
	(−2.06 f)^a^
FVT-2	4.24 ± 0.15^*^
	(−3.50 f)^a^
CT	3.85 ± 0.13*
	(−3.18 f)^a^
AT	2.64 ± 0.075^*^
	(−2.18 f)^a^

^†^Expressed as ratio of HMG-CoA to Mevelonate; lower the ratio higher the enzyme activity.

^*^Values are mean ± SD from liver homogenate of 5 rats in each group.

NC, normal control; HLC, triton induced hyperlipidemic control; FVT-1, fed 50 mg FVBM extract/rat; FVT-2, fed 100 mg FVBM extract/rat; CT, fed 1 mg F18 bioactive compound/rat and AT, given 1 mg atorvastatin/rat for once.

Significantly different from NC at ^a^p < 0.001.

Significantly different from HLC at ^a^p < 0.001.

## Discussion

It is well accepted that enzymes are the major regulators of the lipid metabolism; wherein HMG-CoA reductase is one of the most clinically important enzymes involved in the cholesterol biosynthetic pathway. Changes in the reductase activity are closely related to changes in the overall rate of cholesterol synthesis. This suggests that the inhibition of HMG-CoA reductase would be an effective mean to lower plasma cholesterol [44]. Thus, this enzyme is the target of the widely available cholesterol-lowering drugs known, collectively, as statins [45]. Although, most of the HMG-CoA reductase inhibitors have some adverse side effects [12,13].

In the same vein, oxidative stress also plays a pivotal role in atherosclerosis and subsequent CVD. Therefore, finding an HMG-CoA reductase inhibitor with antioxidant property is of great potential in the treatment and management of hypercholesterolemia and cholesterol induced oxidative stress. The medicinal plants are now being seen as a potent source of the drug discovery program. Epidemiologic studies have shown a correlation between an increased consumption of phenolic antioxidants and a reduced risk of CVD [46-49]. The current interest in combating free radical mediated diseases including atherosclerosis has been shifted toward natural products. The present work was an extension of our recent study in which we screened stem bark and leaf extracts of *Ficus virens* for an HMG-CoA reductase inhibitory activity. Among all the sequentially extracted fractions, FVBM extract showed most potent antioxidant, genoprotective, and HMG-CoA reductase inhibitory activity (IC_50_ value: 3.45 ± 0.45 μg/ml) [20]. In the present study, FVBM extract was subjected to a column chromatography in order to identify and isolate the bioactive compounds as well as to examine their antioxidant and HMG-CoA reductase inhibitor property. Moreover, the role of most potent isolated fraction and the FVBM extract was also investigated in Triton WR-1339 induced hyperlipidemic rats. Twenty fractions (F1-F20) of FVBM extract were collected through column chromatography and screened for antioxidant and HMG-CoA reductase inhibitory activities. DPPH and FRAP methods are frequently used for screening the antioxidant potency of plants [21-23]. DPPH is a stable free radical commonly used to evaluate free radical scavenging activity of antioxidants. The fraction F18 exhibited higher radical scavenging activity of 51% at 5 μg/ml than the other fractions and standard ascorbic acid (12.7% at 5 μg/ml). The ability of this fraction to reduce ferric ions to ferrous ions, generally used to determine the antioxidant power, was also evaluated and it showed significantly higher antioxidant capacity in comparison to other fractions similar to earlier reports [18-20]. As discussed, HMG-CoA reductase is a major regulatory enzyme of cholesterol biosynthetic pathway; and majority of lipid lowering drugs work by inhibiting the activity of this enzyme. Employing a previously standardized protocol [20,43], a marked HMG-CoA reductase inhibitory activity with an IC_50_ value of 84 ± 2.8 ng/ml was observed in fraction F18. Here it is interesting to mention that F18 fraction exhibited better IC_50_ value than that of FVBM crude extract [20]. To the best of our knowledge and on the basis of literature review, the demonstrated HMG-CoA reductase inhibitory activity was significantly better than the reported inhibitors from various plants like kiwifruit [50], *Quercus infectoria, Rosa damascene and Myrtus communis* [51], indicating its potent hypolipidemic property. The fraction F18 was identified as Octadecanyl-O-α-D-glucopyranosyl (6′ → 1″)-O-α-D-glucopyranoside. This is a new and completely different structure than the other inhibitors isolated from various plants such as tocotrienol from barley [52], kakkalide and irisolidone from flower of *Pueraria thunbergiana* [53], camphene, a plant-Derived monoterpene [54], daidzein from *Pueraria thunbergiana* [55], acetylenic acids from the root bark of *Paramacrolobium caeruleum* [56], and flavonoids from the buds of *Rosa damascena* [57].

Furthermore, to understand the binding mechanism and corroborate our *in vitro* hypothesis, *in silico* molecular interaction studies were conducted between HMG-CoA reductase and the inhibitor F18. The docked structure of F18-HMG-CoA reductase involved contributions from Asn^755^, Asp^765^, Lys^691^, Glu^559^, Gly^560^, Ala^525^, Arg^590^ residues, which were not from the catalytic domain of the enzyme. This confirms the uncompetitive mode of inhibition observed *in vitro*.

The result of interaction study of standard drug pravastatin with HMG-CoA reductase is in consensus with our previously published data [20,43,58]. In addition, this novel F18 compound showed good aqueous solubility, low blood–brain-barrier penetration, low intestinal absorption, <90% plasma protein binding, no inhibition effect on cytochrome P_450_ and extremely low hepatotoxicity, indicating it to be safe as a potential drug. In addition, the novel compound also showed weak LDH release which indicates that it is non cytotoxic at concentration of 1 mg.

Based on the above *in vitro* and *in silico* rationale, *in vivo* study was designed to evaluate the hypolipidemic properties of FVBM extract, bioactive compound and standard drug atorvastatin in the triton-induced hyperlipidemic rat model. The triton-induced hyperlipidemic rat model has been successfully employed to evaluate antidyslipidemic activity [59,60]. Triton WR-1339 is a non-ionic surfactant, which elevates the plasma lipids on administration, causes changes in circulatory lipoproteins, suppresses lipoprotein lipase (LPL) activity, hinders the uptake of circulating lipids by extra hepatic tissues, and increases the HMG-CoA reductase activity resulting in increased blood lipid concentrations, hence hyperlipidemia. The present study demonstrated a significant increase in TG, TC, VLDL-C and LDL-C levels with a decrease in HDL-C value in triton-induced hyperlipidemic rats, which was in agreement with previously published data [61]. This increase in plasma TG and TC by triton was due to increase in HMG-CoA reductase activity and by inhibition of lipoprotein lipase responsible for hydrolysis of plasma lipids [60,62]. FVBM extract at higher dose (100 mg/rat) and F18 compound effectively blocked the increase in TG, TC, LDL-C and non-HDL-C levels and reversed them to normal values; thus showed a comparable effect with atorvastatin. Moreover, the HDL-C level was also significantly increased after treatment with the plant extract and F18 compound which in turn offered better protection to LDL as well as HDL from oxidative stress through its associated antioxidant enzyme paraoxonase (PON) [63,64].

It has been established that LDL-C/HDL-C and HDL-C/TC ratios are good predictors of presence and severity of CAD [65]. We observed a significant decrease in HDL-C/LDL-C and HDL-C/TC ratio and increase in TC/HDL-C and LDL-C/HDL-C ratios in hyperlipidemic control rats. The ratios related to TC, LDL-C and HDL-C in FVT-2 & F18 treated groups were positively modulated and restored similar to corresponding ratio values in NC. These results indicate that therapeutic amelioration in plasma TC by F18 or FVBM could be attributed to the changes mainly in LDL-C levels, which emphasize the usefulness of these agents in decreasing cardiovascular morbidity and mortality [66].

The significant increase in plasma and lipoprotein lipid levels in triton induced hyperlipidemic rats is consistent with the increase in hepatic HMG-CoA reductase activity. Earlier studies have demonstrated that intravenous or intraperitoneal injection of Triton WR-1339 increases hepatic cholesterol synthesis by increasing HMG-CoA reductase activity, the rate limiting enzyme in the cholesterol biosynthetic pathway in rodents [67,68]. Treatment of hyperlipidemic rats with FVBM extract (at higher dose) and fraction F18 significantly alleviated the increased lipid levels and restored them close to the normal values, it may provide a mechanism of reduction of plasma cholesterol level in triton induced hyperlipidemic rat model. Our results are in concordance with earlier reports demonstrating that the reduction in HMG-CoA reductase activity is responsible for the hypolipidemic property of natural agents [53,58,69]. This lipid lowering activity might be due to the pleiotropic effect that the compound exhibits through reduced HMG-CoA reductase activity, with significant antioxidant property and increased lipoprotein lipase activity. The compound F18 showed, at an equivalent dose of drug atorvastatin, marked hypolipidemic property and might be a drug candidate.

## Conclusion

In conclusion, our *in vitro, in silico* and *in vivo* results clearly demonstrated the therapeutic efficacy of novel HMG-CoA reductase inhibitor (F18-n-Octadecanyl-O-α-D-glucopyranosyl(6′ → 1″)-O-α-D-glucopyranoside) over statins as a substitute against hyperlipidemia. Moreover, being a natural agent with potent antioxidant and hypolipidemic property, this compound could be used in the protection of ROS/free radical-induced oxidative damage, hyperlipidemia/dyslipidemia and atherosclerotic complications. However, further large scale preclinical and clinical trials are needed to set up its lipid lowering and anti-atherosclerotic properties.

## Additional files

Additional file 1: Figure S1.
^1^H NMR data of fraction F18. Additional file 2: Figure S2.
^13^C NMR data of fraction F18. Additional file 3: Figure S3. IR data of fraction F18. Additional file 4: Figure S4. Mass spectroscopy data of fraction F18.
