# Supplementary figures and images for: Bioactivity guided fractionation and hypolipidemic property of a novel HMG-CoA reductase inhibitor from Ficus virens Ait
Source: Lipids Health Dis. 2015 Mar 4;14:15. doi: 10.1186/s12944-015-0013-6 (PMC4352280; doi:10.1186/s12944-015-0013-6)

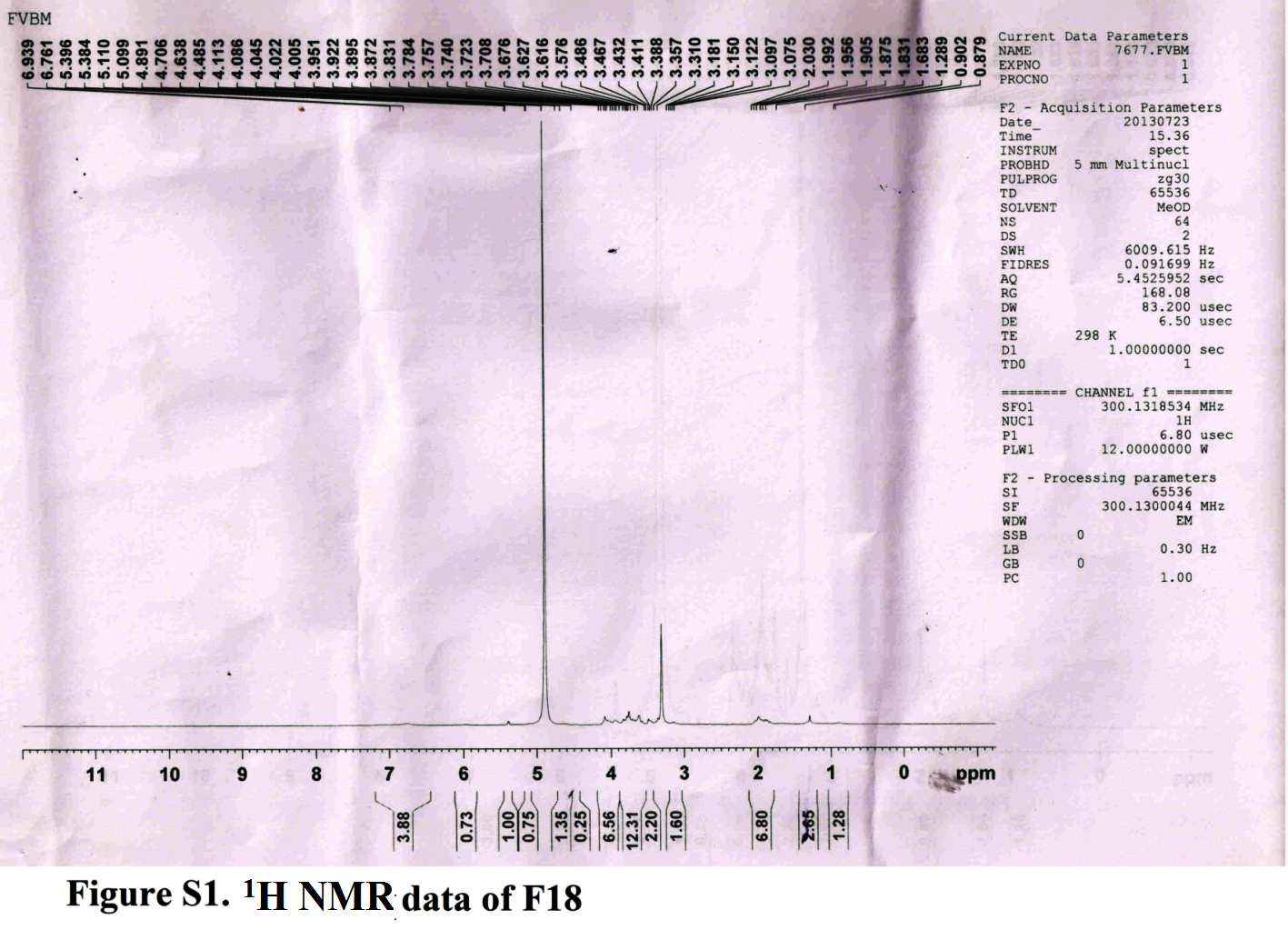

Supplement: Additional file 1: Figure S1. — 1H NMR data of fraction F18. [file 12944_2015_13_MOESM1_ESM.tiff]

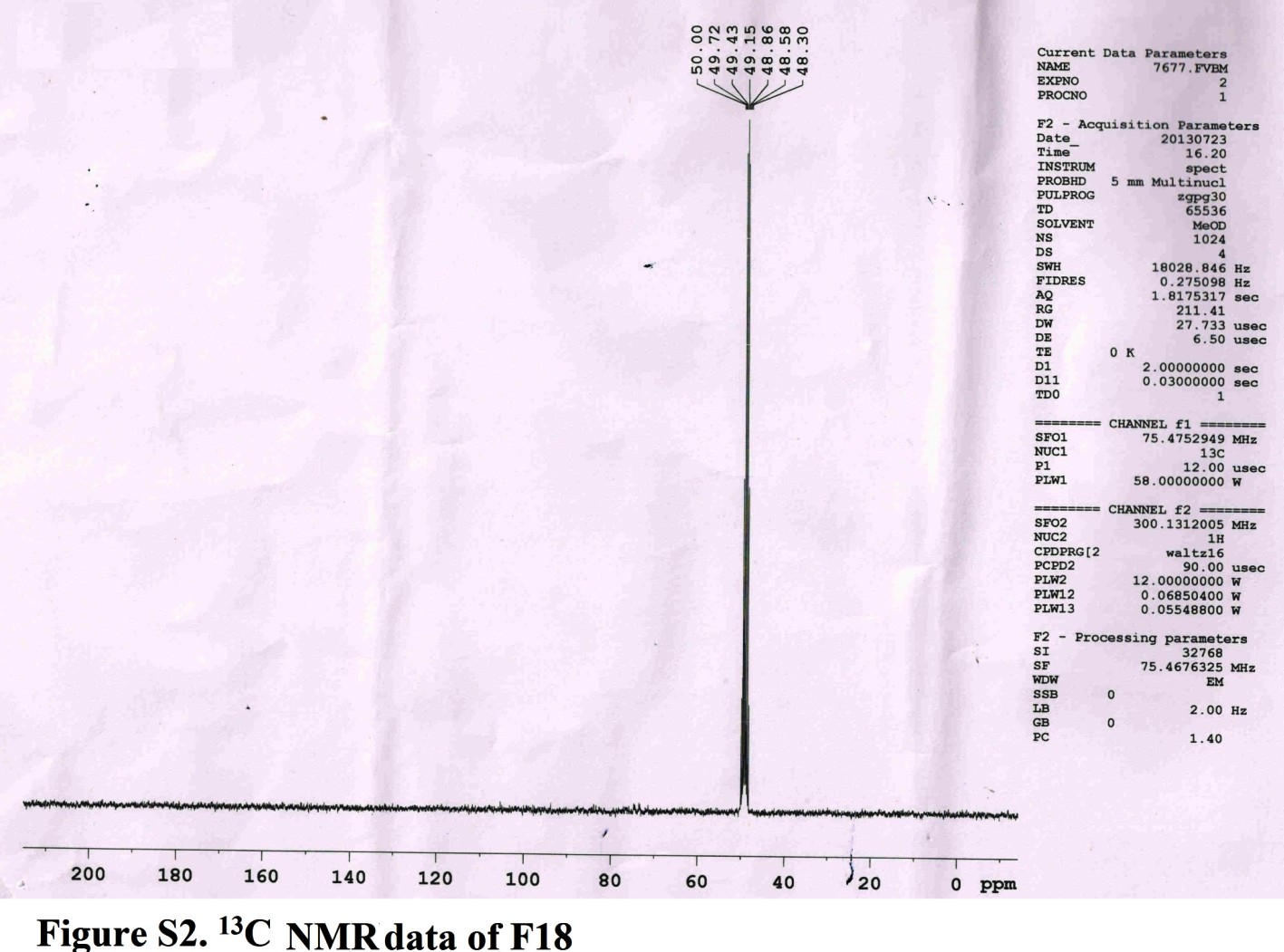

Supplement: Additional file 2: Figure S2. — 13C NMR data of fraction F18. [file 12944_2015_13_MOESM2_ESM.tiff]

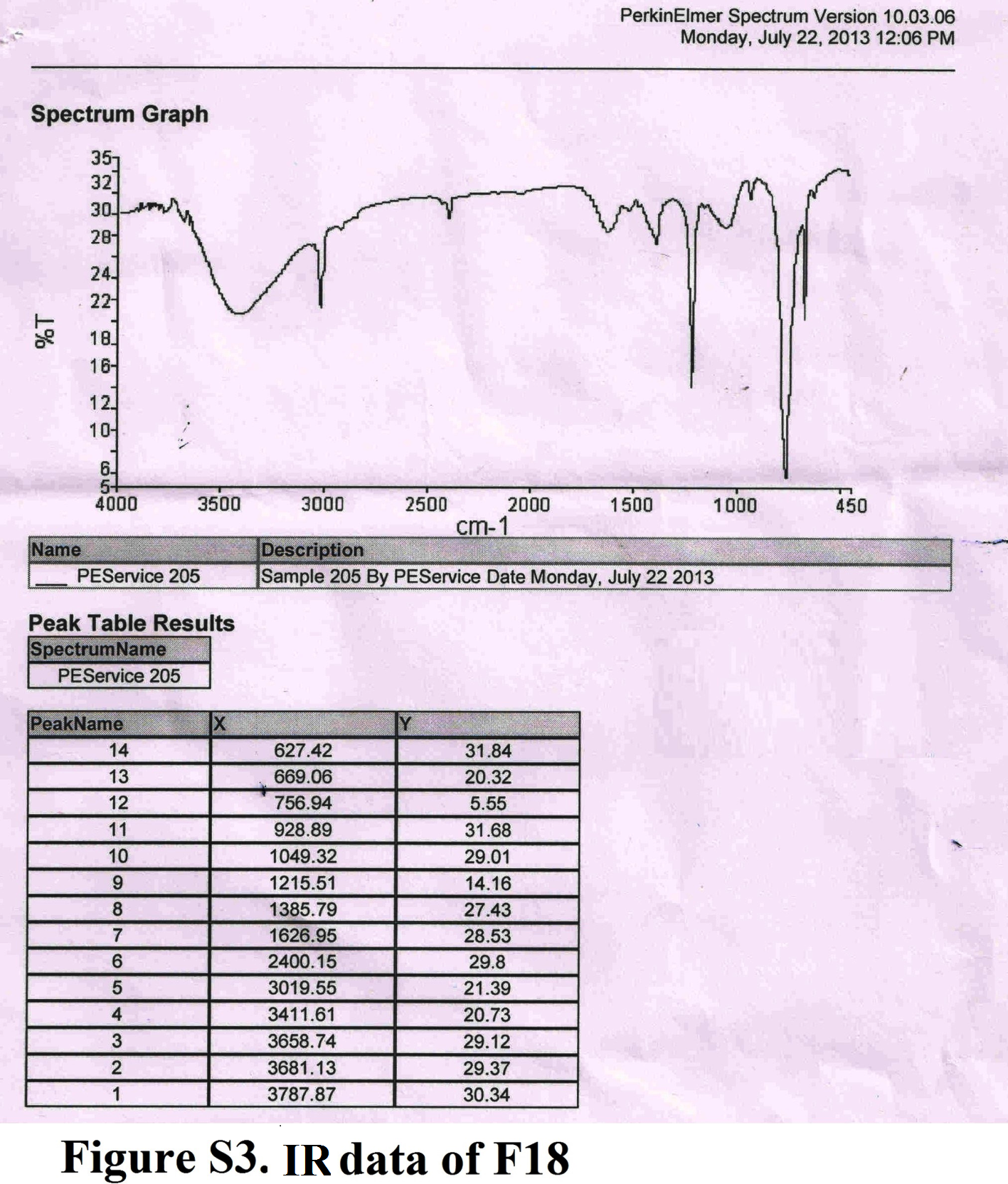

Supplement: Additional file 3: Figure S3. — IR data of fraction F18. [file 12944_2015_13_MOESM3_ESM.tiff]

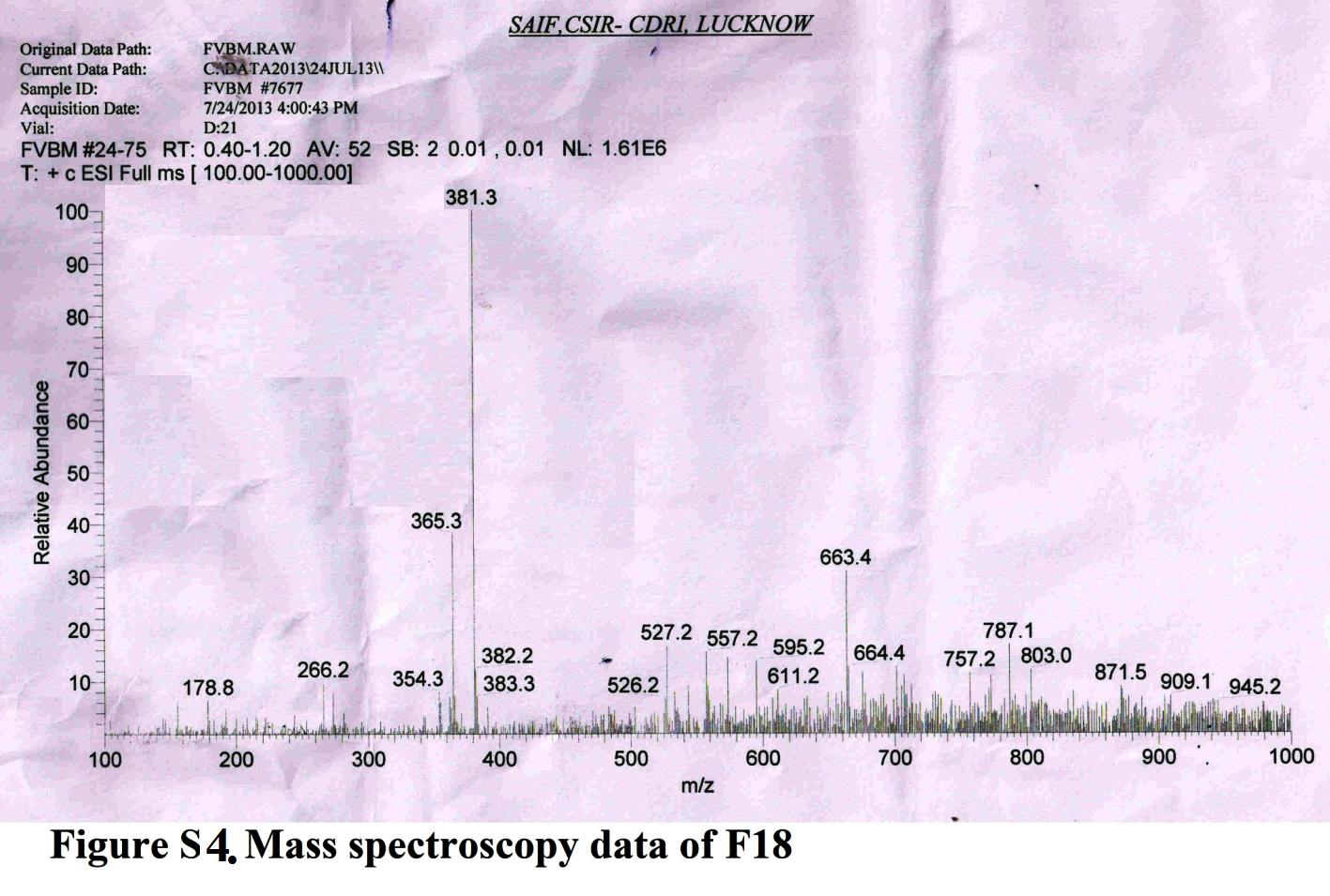

Supplement: Additional file 4: Figure S4. — Mass spectroscopy data of fraction F18. [file 12944_2015_13_MOESM4_ESM.tiff]
